# Wresting SARS from Uncertainty

**DOI:** 10.3201/eid1002.031032

**Published:** 2004-02

**Authors:** Jairam R. Lingappa, L. Clifford McDonald, Patricia Simone, Umesh Parashar

**Affiliations:** * Centers for Disease Control and Prevention, Atlanta, Georgia, USA

**Figure:**
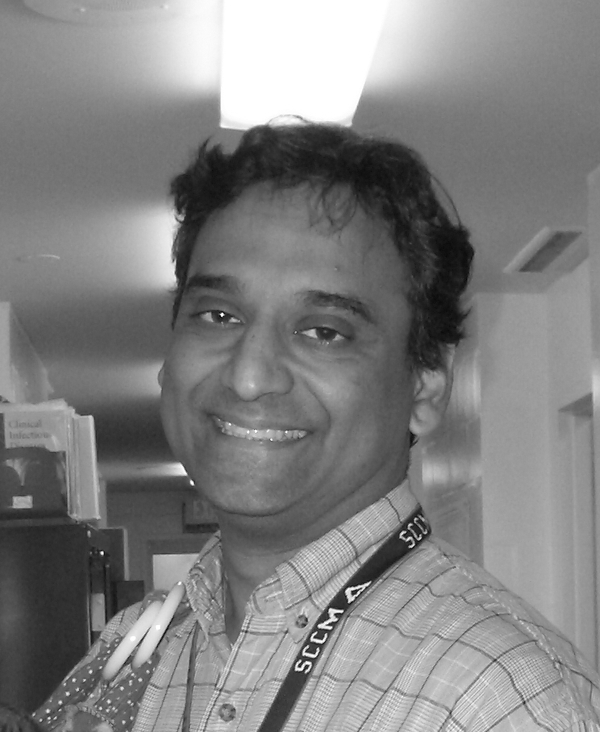
**Dr. Jairam R. Lingappa** worked for the Centers for Disease Control and Prevention (CDC) from 1998 through 2003, most recently as the medical epidemiologist for respiratory viral infections with the Respiratory and Enteric Virus Branch, Division of Viral and Rickettsial Diseases. In that capacity, he had the responsibility for developing epidemiologic evaluation of respiratory viral infections in outbreak settings and research studies. During the 2002–2003 outbreak of severe acute respiratory syndrome (SARS), Dr. Lingappa led the Special Investigations Team coordinating CDC’s SARS transmission and natural history investigations. His research interests include immunologic and genomic aspects of host response to infectious pathogens and vaccines, as well as pathogen detection and discovery technologies and emerging infectious diseases. In January 2004, Dr. Lingappa joined the faculty of the Department of Medicine at the University of Washington.

**Figure:**
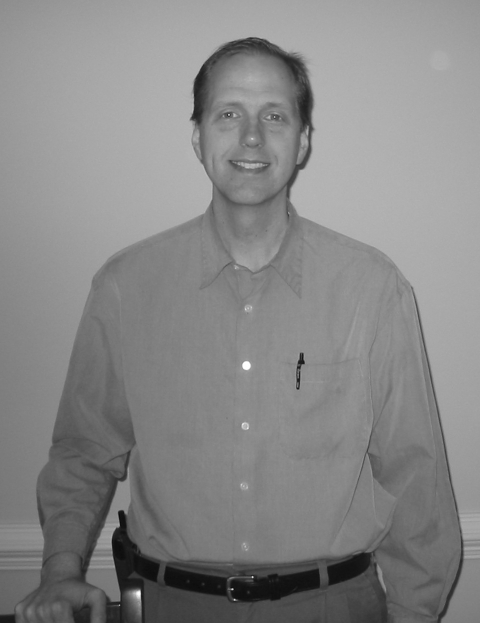
**Dr. L. Clifford McDonald** is a medical epidemiologist in the Epidemiology and Laboratory Branch, Division of Healthcare Quality Promotion, CDC, which has primary responsibility for public health response activities in healthcare settings. Dr. McDonald has training in adult infectious diseases, clinical microbiology, and epidemiology and is an experienced hospital epidemiologist. During the past outbreak, he was a member of the Clinical and Infection Control Team, working in the Emergency Operations Center activated for SARS; he also led the CDC SARS Investigation Team to Toronto during both phases of the outbreak there. His interests include antimicrobial resistance and outbreak investigations in hospitals, and he has performed both domestic and international research in these areas.

**Figure:**
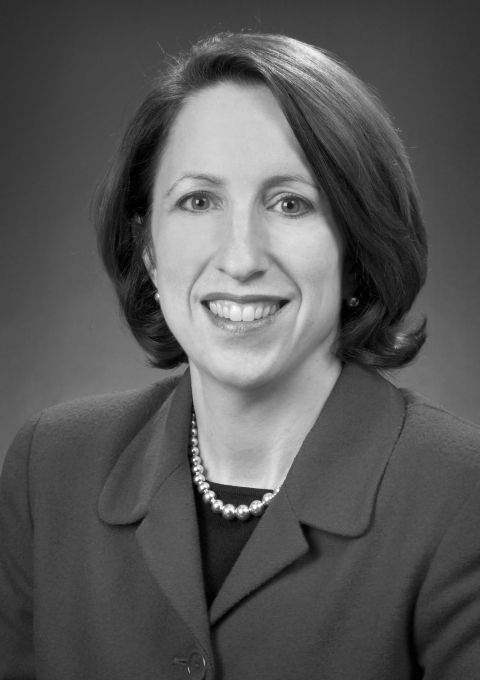
**Dr. Patricia Simone** is the associate director for science in the Division of Global Migration and Quarantine, National Center for Infectious Diseases, CDC. She is responsible for the scientific activities of that division, whose missions are to decrease illness and death from infectious diseases among mobile populations (immigrants, refugees, migrant workers, and international travelers) crossing international borders destined for the United States and to decrease the risk for importation and spread of infectious diseases via humans, animals, and cargo. She is an expert on tuberculosis and serves as the SARS team leader for travel-related issues.

**Figure:**
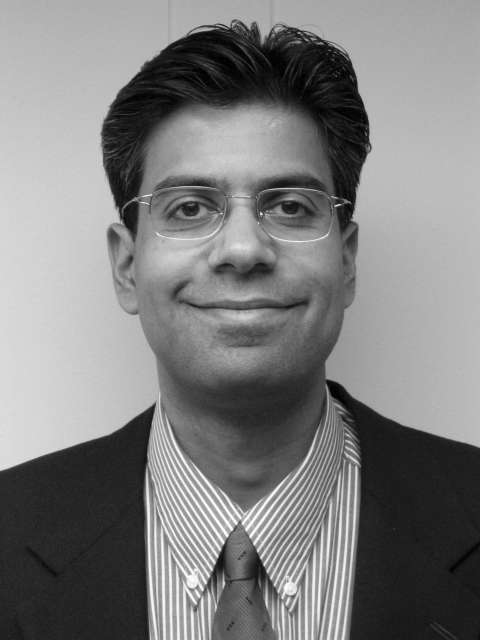
**Dr. Parashar** is the lead medical epidemiologist for the CDC's SARS Task Force, which has overall responsibility to develop, oversee, coordinate, and implement CDC's SARS program activities. Dr. Parashar was a member of the World Health Organization team that investigated the SARS epidemic in Hong Kong and later led the surveillance team at CDC during the response to the SARS outbreak in the United States. His other research interests include the epidemiology of viral gastroenteritis and methods for its prevention and control, including vaccination strategies.

In early March 2003, Dr. Carlo Urbani, a World Health Organization (WHO) epidemiologist stationed in Vietnam, alerted the global health community to the high transmissibility and lethality associated with an apparently new respiratory disease. This disease, now called severe acute respiratory syndrome (SARS), is believed to have emerged in China in November 2002 and progressed to a global health threat by the spring of 2003 (*1*–*3*). On March 15, 2003, with clusters of SARS cases being reported from China, Hong Kong, Vietnam, Singapore, Taiwan, and Canada, WHO issued a global travel alert. At that point, the international health community faced a potential pandemic for which there were no identified causal agent, no diagnostic laboratory assays, no defined properties or risk factors for transmission, no infection-control practices of proven efficacy, and no known treatment or prevention measures. Given that setting, the declaration on July 5 that SARS had been contained (in less than 4 months after its initial recognition), represented a remarkable achievement for a truly extraordinary international public health effort.

However, the SARS outbreak was not contained before it had had a substantial impact: 8,098 cases involving 774 deaths were attributed to SARS (*4*) (the original WHO case definitions [*5*] were revised during the outbreak to those shown in the Table); fear of contagion was rife in many communities, especially among healthcare workers; and billions of dollars had been lost in the airline and tourism industries, resulting in bankruptcies of airlines and other businesses. However, the SARS public health response effort was equally important: the world’s scientific, clinical, and public health communities had successfully instituted sensitive surveillance for the disease; isolation and infection-control practices—with intensive contact tracing and community containment, including quarantine—were effective in limiting continued spread in most cases; and the causative agent and diagnostic assays for detecting the disease were identified.

**Table:** World Health Organization SARS case definitions^a^

**Suspected case-patient**: a person presenting after November 1, 2002,^b^ with a history of (ALL THREE):
1. High fever (>38°C) AND
2. Cough or breathing difficulty, AND
3. One or more of the following exposures during the 10 days before onset of symptoms:
close contact^c^ with a person who is a suspected or probable SARS case-patient;
history of travel to an area with recent local transmission of SARS
residing in an area with recent local transmission of SARS
**Probable case-patient**: a suspected case-patient with:
1. Radiographic evidence of infiltrates consistent with pneumonia or respiratory distress syndrome (RDS) on chest x-ray OR
2. Consistent respiratory illness that is positive for SARS coronavirus by one or more assays, OR
3. Autopsy findings consistent with the pathology of RDS without an identifiable cause

^a^Revised May 1, 2003) (6). SARS, severe acute respiratory syndrome. ^b^The surveillance period begins on November 1, 2002, to capture cases of atypical pneumonia in China now recognized as SARS. International transmission of SARS was first reported in March 2003 for cases with onset in February 2003. ^c^A close contact is someone who cared for, lived with, or had direct contact with respiratory secretions or body fluids of a suspected or probable SARS case-patient.

Now, nearly 1 year after the world first faced this infectious challenge, the public health community is equipped with a broader understanding of the agent, its pathophysiology, clinical signs and symptoms, risk factors for transmission, and public health measures that can successfully contain the disease. The breadth of this understanding and international scope of the outbreak response are reflected in the range of manuscript topics in this issue of Emerging Infectious Diseases. Herein we review some of the salient features of the biology and epidemiology of SARS while underscoring some of the remaining unanswered questions.

The origins of the SARS-associated coronavirus (SARS-CoV) remain unclear; however, data suggest that the outbreak may have been preceded by transmission of this or a related virus from animals to humans. SARS-CoV has now been shown to infect (although not necessarily be transmissible through) other animals, including macaques (*7*), ferrets and cats (*8*), and pigs and chickens (*9*), although none of these animals are known to act as natural amplifying hosts for the virus. Antibodies to SARS-CoV have been identified in animal handlers (*10*), and a SARS-like coronavirus has been identified in palm civets and other animals indigenous to Guangdong Province, where SARS likely originated (*11*). Furthermore, serologic studies in Hong Kong suggest that SARS-like viruses may have circulated in human populations before the 2002–2003 outbreak (*12*).

As the SARS outbreak unfolded in Vietnam, Singapore, and Hong Kong, hospital workers stood out as a critical high-risk group. We now know that in many locations the SARS outbreak began with ill travelers coming from other SARS-affected areas (*13*). For many affected areas with low case numbers, such as the United States (where only eight cases were laboratory-confirmed [*14**–**16*]), SARS remained a travel-associated illness only, with no hospital or community transmission (*14*,*17*,*18*). However, healthcare settings played a key role in amplifying disease outbreaks (*19*). In locations such as Singapore, Canada, and Vietnam, disease was transmitted to many hospital workers by ill travelers or contacts of ill travelers, but in these locations, disease was successfully contained within hospitals. If the disease was not rapidly controlled in healthcare settings, as occurred in China, Taiwan, and Hong Kong, spread into the community occurred, resulting in extensive disease transmission (*20*,*21*) (Figure).

**Figure:**
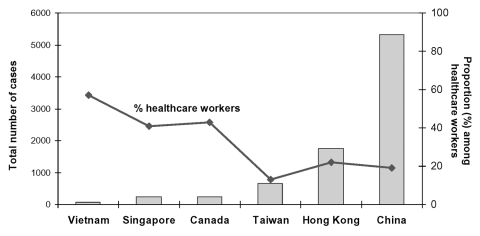
Cumulative cases of severe acute respiratory syndrome and proportion among healthcare workers by geographic region, November 1, 2002–July 31, 2003.

Most SARS patients had a clear history of exposure to other SARS patients or SARS-affected areas. Even in China, despite its extensive community transmission, intensive investigation successfully linked many cases previously classified as “unlinked” to high-risk exposures to SARS patients in fever clinics and other locations (*20*). Older persons were at greatest risk for severe disease, with fatality rates of nearly 50% in persons >60 years of age, whereas, for unclear reasons, fewer children were affected; those that were had lower morbidity and mortality (*22*–*24*).

A critical question has been whether SARS-CoV is transmitted through large droplets or on fomites, as occurs with respiratory syncytial virus, variola, and mycoplasma, or through aerosols, as occurs with measles and varicella. We now know that large droplets are likely the primary mode of transmission; however, in some circumstances, clusters suggestive of aerosol transmission have been described (*19*,*25*,*26*). Transmission appears to be heterogeneous. Most probable SARS cases were associated with little or no transmission. Although low transmission most commonly occurs in association with appropriate infection-control practices (*27*), cases have also been documented with no transmission despite ample exposure opportunities (*17*,*18*,*28*–*30*). Transmission in hospital settings has been clearly documented (*25*,*31*–*33*). Hospital transmission, along with infrequent “superspreading events,” involving transmission from one case to many secondary cases, was critical to propagating the outbreak (*19*,*25*,*26*,*34*). Limited risk factors for superspreading events have been identified, including more severe illness, slightly older age, and an increased number of secondary contacts (*34*); however, further epidemiologic, virologic, and host-factor studies are needed to fully elucidate the risk factors that underlie SARS-CoV transmission.

Fortunately, the outbreak demonstrated that SARS-CoV transmission can be effectively contained by strict adherence to infection-control practices. The use of N95 respirators or surgical masks was found to effectively reduce transmission in hospitals (*31*,*33*); this protective capacity of masks also has been shown for community transmission (*20*). Premature relaxation of infection-control measures in some SARS-affected areas had profound implications (*35*). Studies have demonstrated the importance of preexposure infection-control training and consistent use of masks, gowns, gloves, and eye protection (*36*).

Serologic and nucleic acid assays to detect SARS-CoV infection and virus, respectively, were developed early in the outbreak investigation (*37*–*39*). Comparative studies have now confirmed the sensitivity and specificity of enzyme-linked immunosorbent assays for detecting SARS antibodies (*40*) and of multitargeted real-time reverse transcription–polymerase chain reaction (RT-PCR) assays for detecting SARS-CoV infection (*41*,*42*). Although these assays are sensitive for detecting antibody and viral RNA, they have provided limited help in diagnosing SARS early in the course of disease (*15*,*16*,*43*,*44*). However, since the SARS clinical case definition is nonspecific, capturing respiratory illness caused by other pathogens (e.g., *Mycoplasma pneumoniae* and influenza) (*14*), laboratory confirmation of SARS-CoV infection is of particular importance for focusing control efforts during an outbreak and for refining SARS clinical studies. Such studies have shown that less than one third of patients initially have respiratory symptoms and, although abnormal findings on chest radiographs are universal for SARS patients, radiographic changes may not be discerned until 7 to 10 days after illness onset (*45*,*46*).

Diagnostic assays have also been important in describing the natural history of SARS infection and the associated immune response (*29*,*43*,*47*). Seroconversion within 28 days after symptom onset has been documented in 92% to 100% of probable SARS cases. Furthermore, during the first 4 days of illness, SARS-CoV is detectable by RT-PCR in respiratory secretions from less than half of the case-patients. Virus is subsequently detected in stool, and peak levels in both respiratory and stool specimens are found by day 11–12 of illness; virus can persist in stool for weeks thereafter (*29**,**42**,**43**,**47*). These studies underscore the continued need for SARS-CoV laboratory assays that are sensitive early in the disease course to support rapid clinical and infection-control decision-making.

The possibility remains that SARS may reemerge from unidentified animal reservoirs or from persistently infected humans. Current planning efforts for response to a future SARS resurgence rely upon vigilant application of clinical and epidemiologic criteria to evaluate cases of febrile illness (*48*). A bold and swift public health response to this disease must be applied with fairness and in a manner that preserves dignity for all. Response to any future resurgence of SARS will be aided by the body of knowledge about the infection that now exists and by the international experience in successfully containing the first SARS outbreak.
